# Necrophagy by insects in *Oculudentavis* and other lizard body fossils preserved in Cretaceous amber

**DOI:** 10.1038/s41598-023-29612-x

**Published:** 2023-02-18

**Authors:** Mónica M. Solórzano‑Kraemer, Enrique Peñalver, Mélanie C. M. Herbert, Xavier Delclòs, Brian V. Brown, Nyi Nyi Aung, Adolf M. Peretti

**Affiliations:** 1 Senckenberg Research Institute and Natural History Museum, Senckenberganlage 25, 60325 Frankfurt Am Main, Germany; 2 CN-Instituto Geológico y Minero de España CSIC, C/Cirilo Amorós 42, 46004 Valencia, Spain; 3 Departament de Dinàmica de la Terra i de l’Oceà, Faculty of Earth Sciences, 08028 Barcelona, Spain; 4 Institut de Recerca de la Biodiversitat (IRBio), Universitat de Barcelona, 08028 Barcelona, Spain; 5 Entomology Section, Natural History Museum of Los Angeles County, 900 Exposition Boulevard, 90007 Los Angeles, CA USA; 6 Myanmar Geosciences Society, c/o Department of Geology, University of Yangon, 11041 Yangon, Myanmar; 7 Peretti Museum Foundation, Baumschulweg 13, 6045 Meggen, Switzerland; 8 GRS Gemresearch Swisslab AG, Baumschulweg 13, 6045 Meggen, Switzerland

**Keywords:** Ecology, Behavioural ecology, Biodiversity, Evolutionary ecology, Palaeoecology

## Abstract

When a vertebrate carcass begins its decay in terrestrial environments, a succession of different necrophagous arthropod species, mainly insects, are attracted. Trophic aspects of the Mesozoic environments are of great comparative interest, to understand similarities and differences with extant counterparts. Here, we comprehensively study several exceptional Cretaceous amber pieces, in order to determine the early necrophagy by insects (flies in our case) on lizard specimens, ca. 99 Ma old. To obtain well-supported palaeoecological data from our amber assemblages, special attention has been paid in the analysis of the taphonomy, succession (stratigraphy), and content of the different amber layers, originally resin flows. In this respect, we revisited the concept of syninclusion, establishing two categories to make the palaeoecological inferences more accurate: eusyninclusions and parasyninclusions. We observe that resin acted as a “necrophagous trap”. The lack of dipteran larvae and the presence of phorid flies indicates decay was in an early stage when the process was recorded. Similar patterns to those in our Cretaceous cases have been observed in Miocene ambers and actualistic experiments using sticky traps, which also act as “necrophagous traps”; for example, we observed that flies were indicative of the early necrophagous stage, but also ants. In contrast, the absence of ants in our Late Cretaceous cases confirms the rareness of ants during the Cretaceous and suggests that early ants lacked this trophic strategy, possibly related to their sociability and recruitment foraging strategies, which developed later in the dimensions we know them today. This situation potentially made necrophagy by insects less efficient in the Mesozoic.

## Introduction

The decay of vertebrate carcasses in terrestrial environments, from death to the dispersion of the bone, is accompanied by sequential assemblages of necrophagous arthropod species, most of them insects^1^. These insects that feed on the available nutrients exhibit some specialization depending on the geographic area, the surrounding environment, and the size of the carrion^2^. The complex succession of arthropods visiting decomposing carcasses, to eat and/or to lay eggs, as well as the study of the development of the immatures that also feed on carcasses is a key topic in forensic studies^3^. Examples of these trophic events are elusive in the fossil record, but a few cases are known showing evidence of one stage of the succession, in the Mesozoic^4,5^ and Cenozoic^6,7^, but apparently are lacking for the Palaeozoic^8^. Most of these examples are related to exceptional preservation, in which the carcass is preserved as evidence of a certain degree of decomposition, in combination with remains of the necrophagous arthropods or evidence of their activity.

Amber is an example of exceptional preservation of fossils, and examples of ‘frozen behaviour’(“*rare, unusual, but coevolutionary and behaviorally critical specimens in which an organism(s) is preserved while actually doing something*”, sensu Boucot^9^) are known from it^10^. Cretaceous amber could provide interesting evidence of trophic behaviours in forests during the Mesozoic. However, the study of this kind of evidence requires accurate taphonomical analyses. Understanding the entrapment processes of organisms by resin, in general, and the reconstruction of the processes that occurred in the origin of an amber piece containing bioinclusions (specimens of organisms or their remains included in amber, copal and Defaunation resin), in particular, is key for the interpretation of amber assemblages, and thus for the reconstruction of resinous forest palaeoenvironments^11–13^. Determining the main taphonomical biases is essential to understand the structure of ancient food webs, which, in turn, is also essential to identify keystone species within the trophic structure of a community in the past and the present^14,15^. One key is the process by which nutrients tied up in dead animals of various sizes are released by the activity of scavenger animals^16,17^. Dipterans are well known as decomposers of vertebrate carcasses, playing an essential role in the forests^18–21^ and are also abundant as bioinclusions in resin^12,22,23^. In resiniferous forests, the relevance of a precise analysis of which kinds of organisms are trapped in the resin and which are not (or with an extremely low possibility of becoming trapped) was studied by Solórzano Kramer et al.^12^. Single amber pieces with more than one inclusion have been studied with the goal of understanding some aspects of the structure of ancient food webs^15,24,25^. The description of single specimens is undoubtedly the most crucial task to identify diversity patterns^26^, yet information about their fossilization processes and their environments can complement the picture.

Each fossil specimen in an amber piece containing more than one is called syninclusion. The syninclusion concept was discussed by Koteja^27,28^, but the definition of the term syninclusion was created later^29^ as “specimens embedded in the same amber piece”. This definition considers as specimens only organismal elements, such as animal bodies or remains of animals, plants and microorganisms, and excludes sap pseudoinclusions (secreted by the resinous trees together with the resin)^30^, for example. Also excluded are inorganic particles of soil or bubbles. Syninclusions are fossilized organisms, or parts of them, present in a single amber piece and thus trapped in the original resin at very close proximity and approximately at the same time. In most cases they were trapped simultaneously or within minutes, hours or days of difference. Such a set of syninclusions can give accurate information about the different faunistic elements that were coexisting in a restricted area^25,31^. Also, they can provide direct evidence of behaviour or interactions between organisms, both intra- and interspecific, which could be key to understanding relevant evolutionary aspects^32–36^, and not simply data of the taphocoenosis (assemblage of dead organisms) or oryctocenosis (part of a taphocoenosis that has been preserved as a fossil) from a single amber piece. With the help of the data provided by sets of syninclusions from a large number of amber pieces, an accurate, well-supported reconstruction of the resiniferous forested habitat can be accomplished^29^. Syninclusions must be studied in detail and using diverse taphonomic evidence, including comparisons with actualistic observations in nature or in experiments.

Here, we present a detailed study on the resin layers of an amber piece containing the lizard *Oculudentavis naga* Bolet et al., 2021 and other bioinclusions. Furthermore, other exceptional Cretaceous amber pieces were examined to obtain well-supported palaeoecological conclusions of the same trophic topic to be contrasted with Miocene amber and actualistic data.

## Results

### Syninclusions: eusyninclusions and parasyninclusions

We propose here the new terms eusyninclusions and parasyninclusions. The concept of syninclusions (“*Syninclusions are specimens embedded in the same amber piece. It is the only evidence that two or more organism lived in the same time and site.*”^29^) is mostly used in palaeoentomology, and is virtually absent in palaeobotany and vertebrate palaeontology. It has been proved its notable utility in amber studies. However, the study of amber pieces having abundant syninclusions with consideration of the different layers (resin flows; each can be considered a recorded microevent) and their sequence in time is not usually done. Although, the results of the study of each layer separately can strengthen the plausibility of the proposed biological relationships and behaviours^37,38^. Here we distinguish and defined two kinds of syninclusions: (1) *eusyninclusions*: prefix (eu-) in its sense “true”- bioinclusions present in a single layer of an amber piece, and (2) *parasyninclusions*: prefix (para-) in its sense “alongside”- bioinclusions present in different layers of an amber piece in respect to a defined layer that contains eusyninclusions by definition. We consider here the term defined by Koteja^29^ as syninclusions *s.l.* If the different contents of different layers are not discriminated, not considered separately, then the bioinclusions of an amber piece can be named either syninclusions *s.l.* or parasyninclusions. One amber piece with more than one bioinclusion and that only records material from an original resin flow, or records more than one original resin flow but all the bioinclusions are present in one of them, then only contains eusyninclusions (in these cases, better the use of “eusyninclusions” instead of “syninclusions *s.l.*” or “bioinclusions”, because is more restrictive and thus more informative).

### Description of the amber piece GRS-Ref-28627

**Figure 1:**
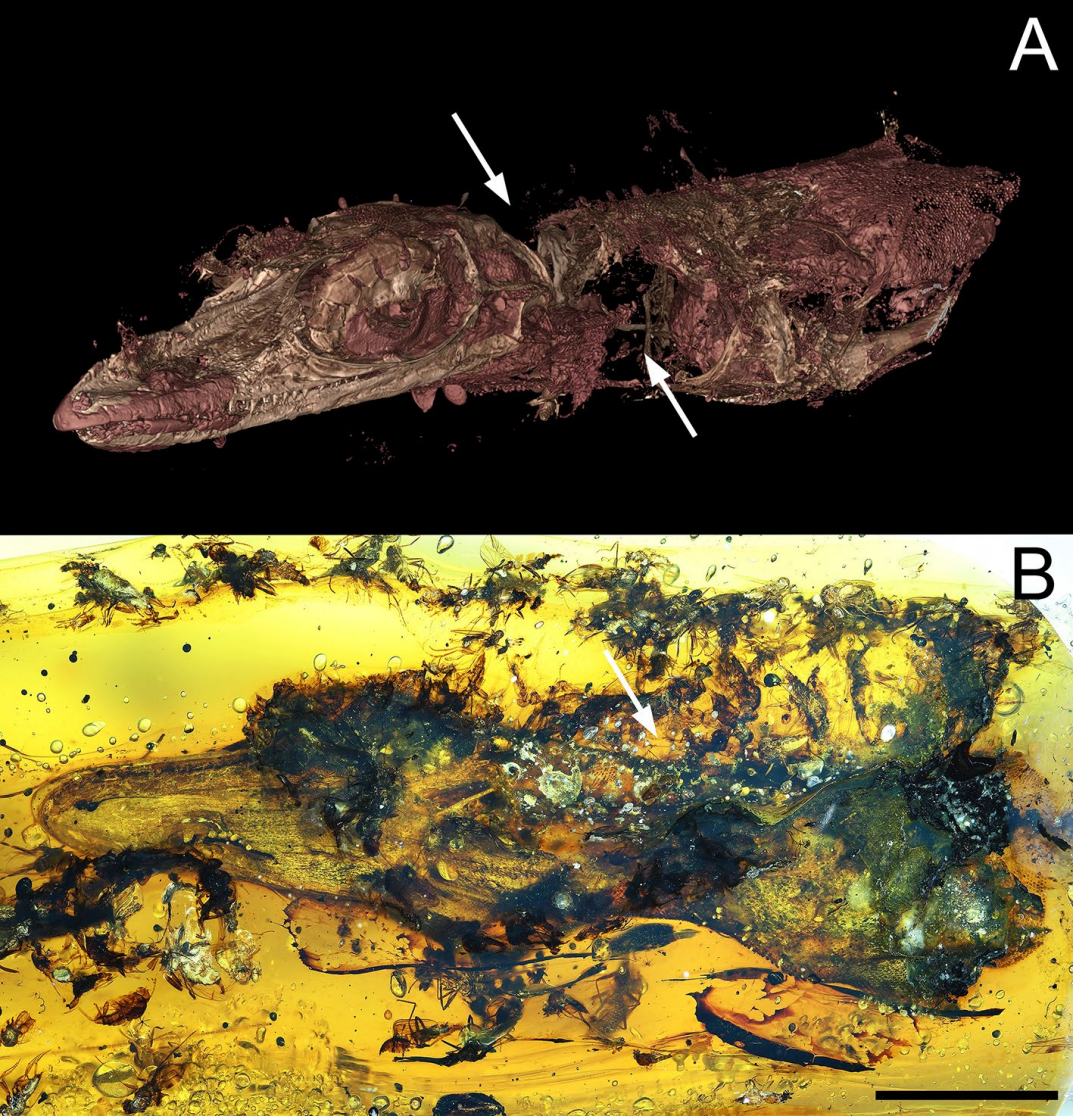
Piece GRS-Ref-28627 with *Oculudentavis naga* Bolet et al.^39^. (**A**) Virtual representation of *Oculudentavis* in frontolateral view (arrows show the place where the soft tissues were already partially consumed). (**B**) Photograph of *Oculudentavis* in ventrolateral view (arrow shows the place where most of the flies were trapped into the resin), as observed in side A. Scale bar 1 mm. Arnau Bolet provided high resolution photo for A.

The analysed amber piece is about 5.2 cm long and contains more than 130 animal bioinclusions and plant trichomes. The most prominent one is the holotype of *Oculudentavis naga*. This species and *Oculudentavis khaungraae* Xing et al., 2020 were classified within Squamata^39^, one of the biggest orders containing lizards, snakes and amphisbaenians or worm lizards. Bolet et al.^39^ focused on the description of the new taxon, but not on the diverse data that the abundant syninclusions of the piece can provide. One interesting bioinclusion is a large beetle probably of the superfamily Elateroidea. Elateroidea, especially species belonging to the family Elateridae, are abundant in Burmese (Myanmar) amber^40^. The amber piece shows 13 resin layers recorded (Figs. 1, 4). The layers of interest containing *Oculudentavis* and the Elateroidea beetle are the number 12 and the number 10, respectively, and are separated by a thin layer (number 11) only containing a few bioinclusions.

Besides the lizard and the large beetle, we noticed that two fly morphotypes are extremely abundant, making up more than 90% of the bioinclusions in the amber piece (Figs. 1B, 3A,C,E). One of them belongs to the family Phoridae, in the genus *Prioriphora*, according to the unpublished work of one of the authors (M.C.M.H.) (Fig. 3D), who is revising the Cretaceous amber phorid fauna. These phorids have been considered in a single morphotype, but without total certainty; the preservation of the flies is not homogeneous, and some of them are in bad shape, so more than one morphotype (more than one species?) could be involved. The second one belongs to the superfamily Empidoidea, probably to the family Atelestidae; however, it will be hereafter called Empidoidea morphotype 1 (Fig. 3B), because no taxonomical revision is proposed here. The other two morphotypes of Empidoidea dipterans, represented only by a few specimens, are also eusyninclusions with *Oculudentavis*, and to the Elateroidea beetle, hereafter called morphotypes 2 and 3 (Fig. 3F,G).

**Figure 2:**
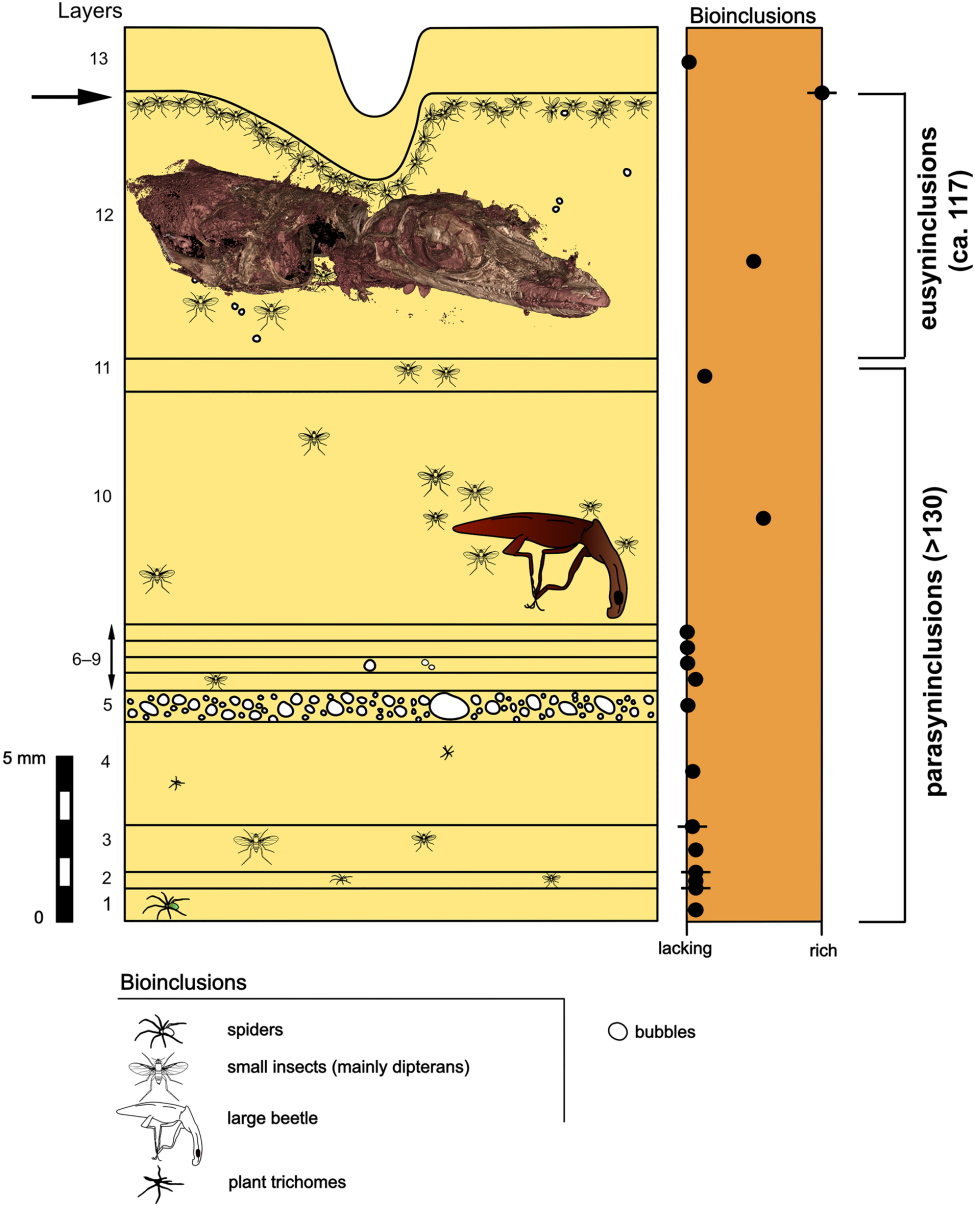
Stratigraphy showing the layers (resin flows) preserved in the piece GRS-Ref-28627 and their contents in a very schematic manner with indication of the different significance, specific for our case of study, of the new terms eusyninclusions and parasyninclusions. The relative abundance in bioinclusions is represented at the right for each whole layer, except to for four layers in which there two indications, the relative quantity of bioinclusions in the top surface as striped points and the remained relative quantity of bioinclusions as a point at the middle of the layer; arrow indicates the interesting surface plenty of necrophagous dipterans, which is the top of the layer 12 containing the *Oculudentavis naga* holotype and a large portion of wings of a neuropteran individual. The large beetle most likely is an Elateroidea. The thicknesses of the layers represented are the greater observed, but they vary along the two apices of the piece and also as observed in their contacts with the two surfaces of the piece in sides A and B (these sides are illustrated in Fig. 4A—the side A- and Fig. 4C—the side B-); original thicknesses of layers 1 and 13 greater and represented as preserved. The taphonomy of the piece (mostly of the layers 12 and 13) stablished the chronology from the base (layer 1, the oldest) to the top (layer 13, the younger). Silhouettes of the bioinclusions and bubbles not to the same scale. Produced using Affinity Designer (www.affinity.serif.com/de/designer/) and Adobe Photoshop (www.adobe.com).

**Figure 3:**
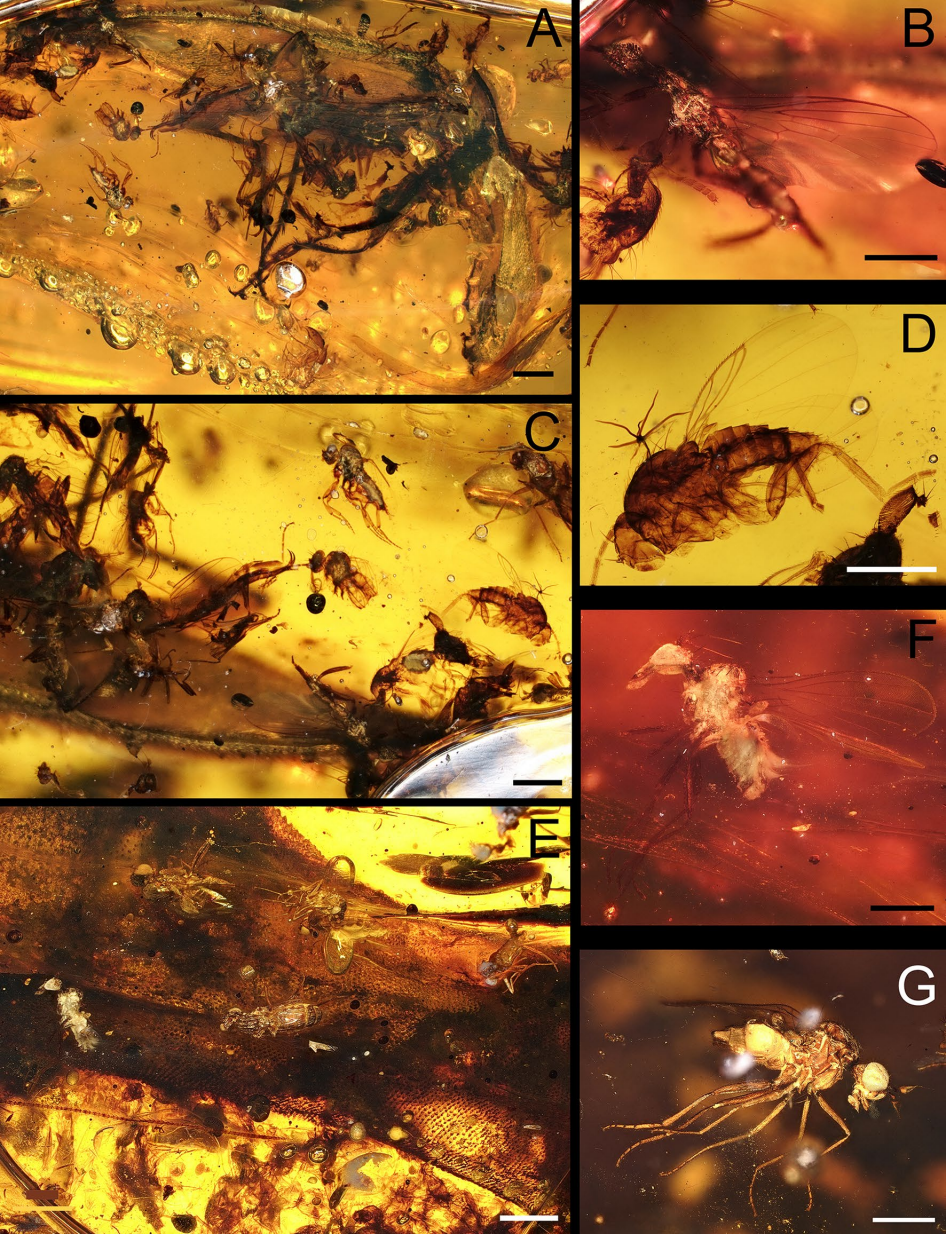
Syninclusions s.l. in piece GRS-Ref-28627 being the most relevant the holotype of *Oculudentavis naga* and the beetle, most likely an Elateroidea. (**A**) Elateroid beetle. (**B**) Empidoid fly morphotype 1. (**C**) Group of flies as eusyninclusions with the Elateroidea beetle. (**D**) Phorid fly. (**E**) Group of flies as eusyninclusions with *O. naga*. (**F**) Empidoid fly morphotype 2. (**G**) Empidoid fly morphotype 3. Scale bars: (**A**, **C**, and **E**) 1 mm, (**B**, **D**, **F**, and **G**) 0.5 mm.

**Figure 4:**
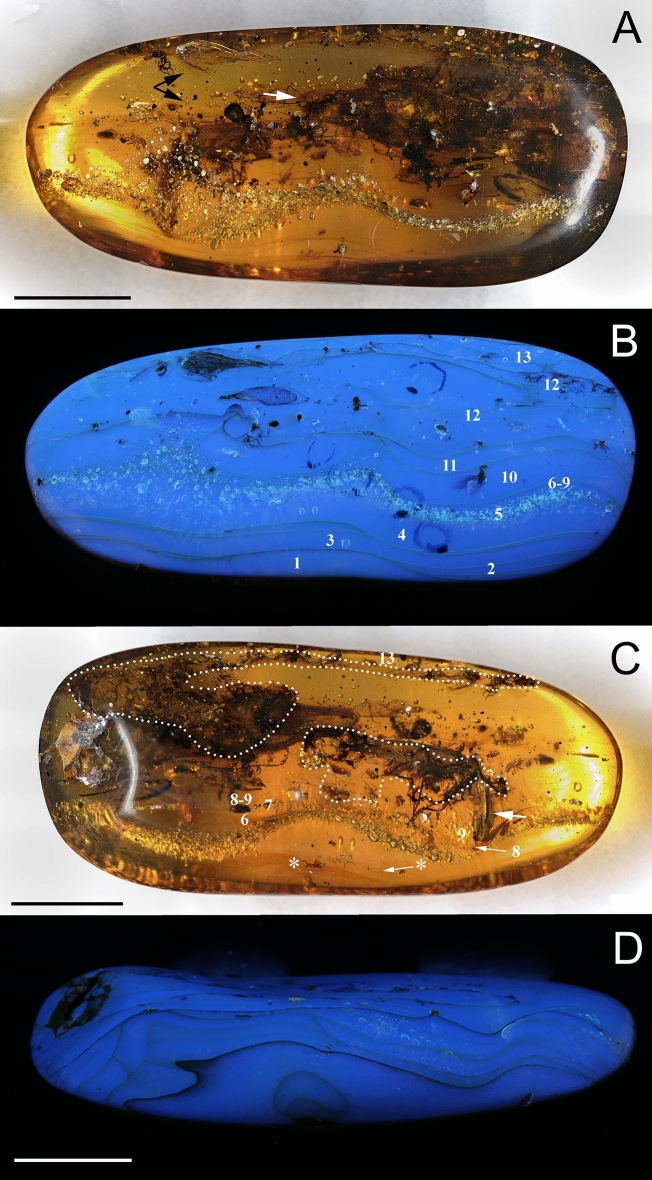
Piece GRS-Ref-28627 photographed with visible light and UV light. (**A**, **B**) Layers with their numeration (see Fig. 2) and main syninclusions s.l. as observed in the piece side A (in A: the two black arrows indicate the large portions of a neuropteran individual and white arrow indicates the tip of the rostrum of the *Oculudentavis naga* holotype). (**C**) Layers with their numeration (see Fig. 2) and bioinclusions as observed in the piece side B; the two areas preserved of the top surface of layer 12 (surface of interest) are marked with dotted lines (white arrow indicates the head of the large beetle, most likely an Elateroidea; asterisks indicate the darker top surfaces of the layers 1 and 2). (**D**) UV light photograph of the piece in lateral view showing the limits of some layers. Images (**A**) and (**B**) to the same scale and orientation. Scale bars 1 cm.

Layers 1–3, 6 and 11 contain other syninclusions as such: five Diptera (one indeterminate, one individual of a Ceratopogonidae, one individual of a Sciaroidea, one individual of a Chironomidae, and individual of another Empidoidea), one Psocodea, one nymphal Orthoptera, one Hymenoptera, the wing of a Neuroptera, two Araneae, three Coleoptera beside the large Elateroidea, and two antennae of an indeterminate insect. Furthermore, plant trichomes are also present in layer 4. The determination at different taxonomic levels is due to the preservation of the bioinclusions and the particular focus on Diptera. Layer 12 contains most of the flies of the Empidoidea morphotypes 1–3 and Phoridae as eusyninclusions (Fig. 3B,D,F,G), which are around *Oculudentavis*'s neck (Figs. 1, 2). Layer 10 contains the large beetle with flies of Empidoidea (morphotype 1 and 3) and Phoridae as eusyninclusions in this set (Figs. 3B,D,G, 4).

### Similar patterns in other amber pieces from Myanmar and in actualistic experiments

**Figure 5:**
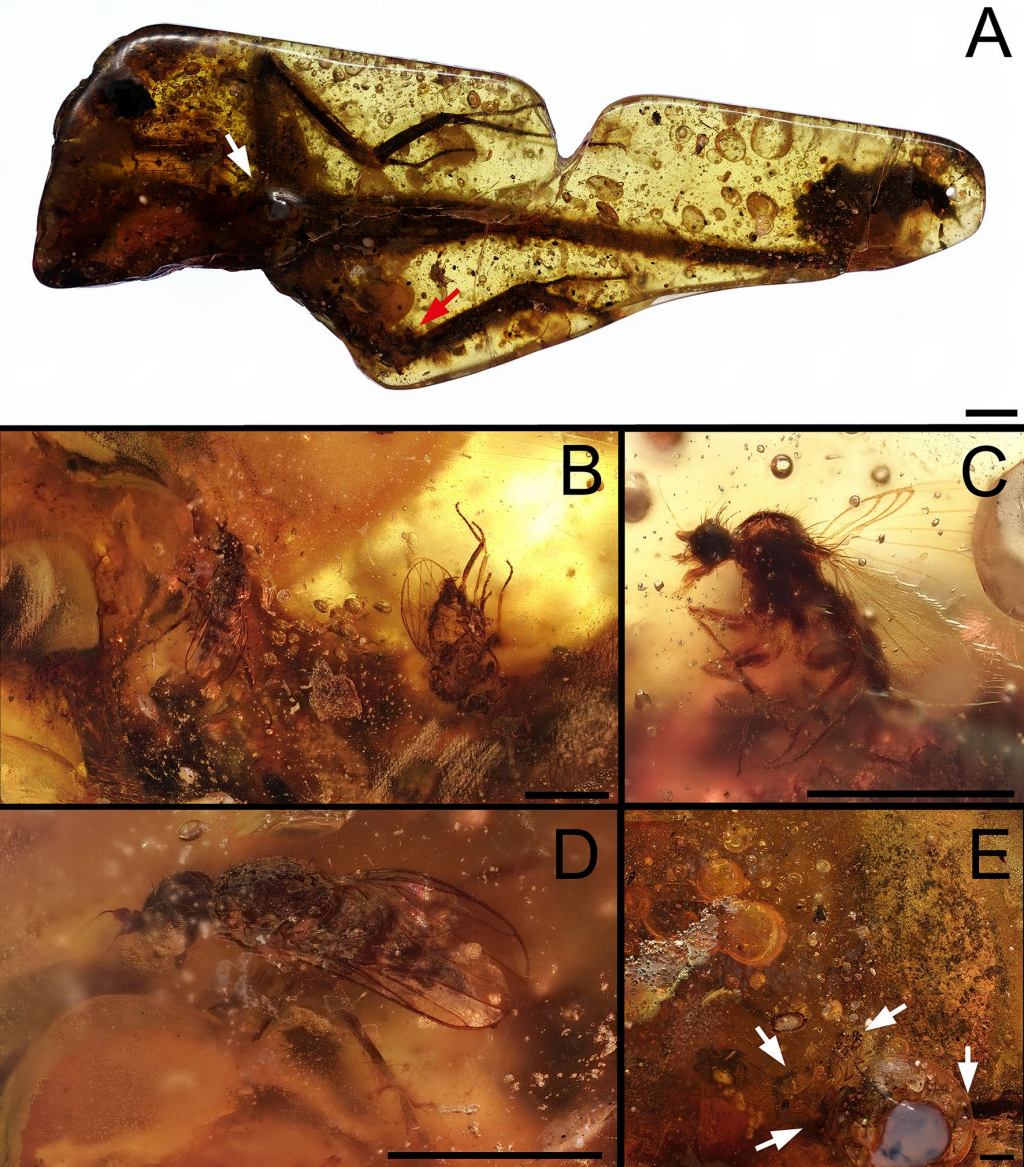
Piece number GRS-Ref-28631 with an incomplete lizard, probably of the family Agamidae. (**A**) Habitus of the lizard (white and red arrows show the place where the flies are located). (**B**) Two Empidoidea. (**C**) One Phoridae. (**D**) One Empidoidea. (**E**) Other Phoridae (white arrows show the position of the flies, which are not easy to observe in this dark amber piece). Scale bars: (**A**) 5 mm, (**B**–**E**) 1 mm.

**Figure 6:**
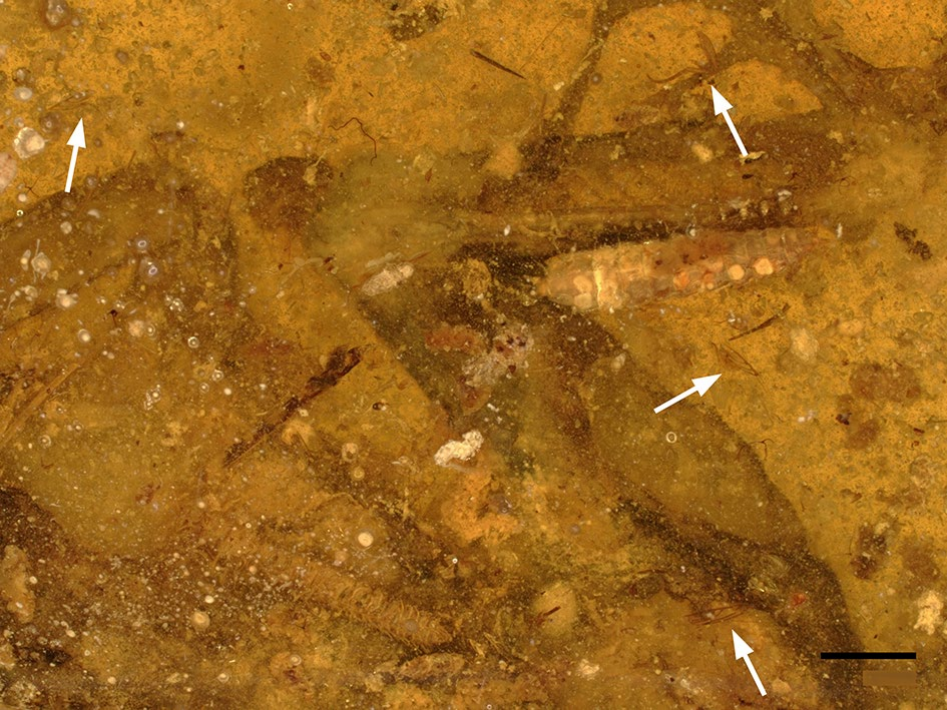
Amber piece Number GRS-Ref-060905. The Picture shows the ventral, left side of a lizard portion, primarily the hind leg, probably of the family Agamidae. Four white arrows show wings and legs of flies of the family Phoridae. Scale bar 1 mm.

Phorids are generally diverse in Cretaceous amber as a whole but in Burmese amber are specially highly diverse (unpublished work of M.C.M.H.). However, as far as we could observe and without a taxonomic description, the morphotypes in pieces GRS-Ref-28627, GRS-Ref-28631, and GRS-Ref-060905 are the same or highly similar to those in layers 10 and 12 above described. Empidoidea and Phoridae, around the fossils of agamid lizards^41^, are observed in the pieces GRS-Ref-28631 (Fig. 5) and GRS-Ref-060905 (Fig. 6). Principally, the family Phoridae is highly abundant. Also, the pieces GRS-Ref-32148, GRS-Ref-28633, GRS-Ref-28617 (Peretti Museum Foundation collection), which contain Squamata (lizards), contain Phoridae as syninclusions; however, these in each piece are not in the same layer, and are considered here neither to be related to the presence of the lizards, nor to be necrophagous. The coincidence of these flies very close the lizard bodies, but in a different layer, could be explained by the release of molecules from the carcasses through the fine cover of fluid resin, which attracted a few of them. Some lizards show a kind of emulsion, a *post mortem* reaction, around their bodies that with certainty was caused also by some degree of decomposition within the resin as it has been observed in other bioinclusions in amber^42^. In any case, we mention these herein as interesting study cases for potential further taxonomical works in order to determine these phorids and know if they belong to the same taxon.

**Figure 7:**
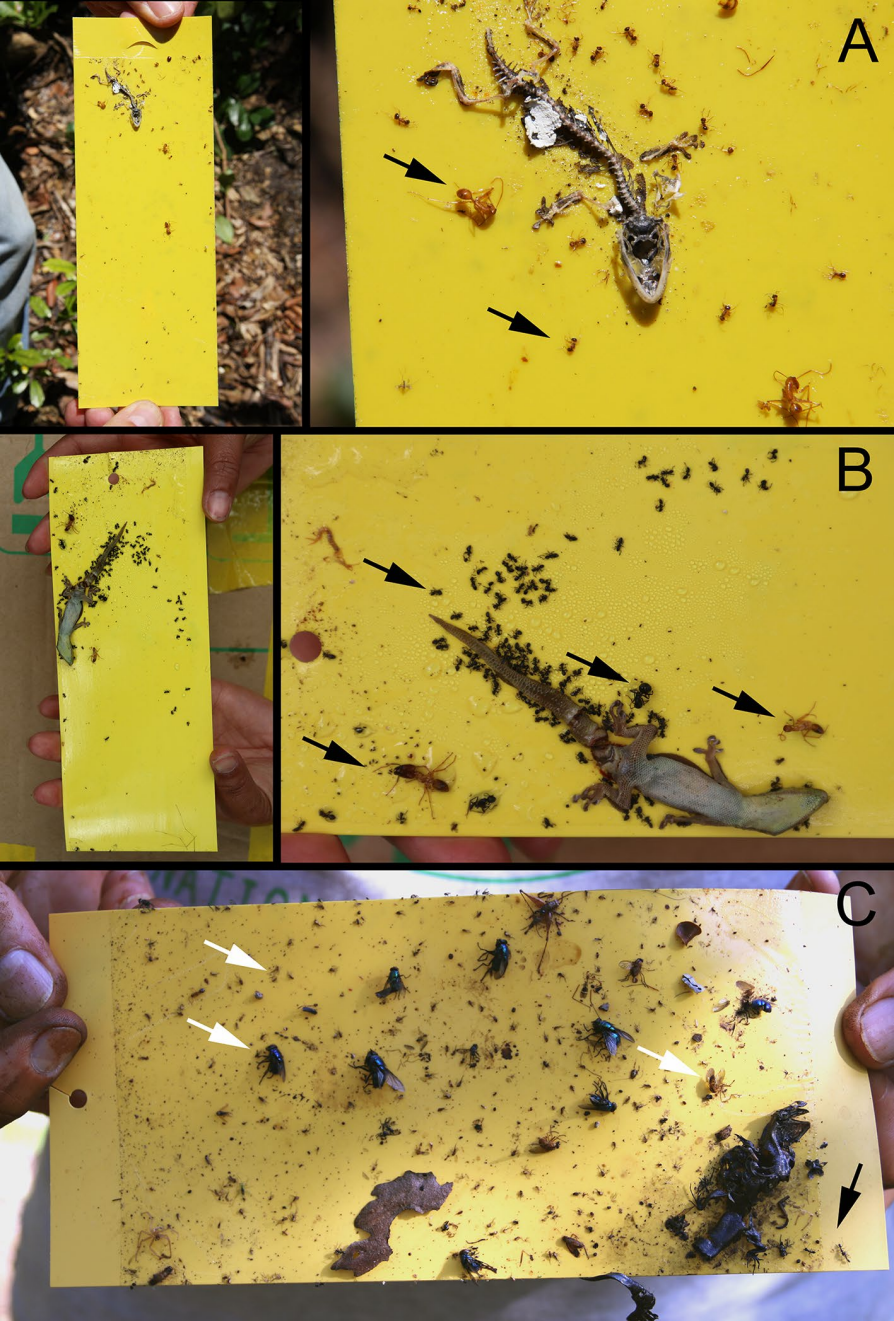
Extant taphonomic correlates from actualistic field research in Madagascar and New Caledonia using yellow sticky traps on trunks of the resinous tree species *Hymenaea verrucosa* (Fabaceae) and *Agathis lanceolata* (Araucariaceae), respectively. (**A**) Gecko trapped on sticky trap placed on the tree H4 at 2 m height on the *Hymenaea* trunk; note abundant specimens of two ant morphotypes (two black arrows) of different size which became trapped from the numerous ants that detected the gecko carcass and skeletonized it. (**B**) Gecko trapped on sticky trap placed on the tree 3R2 at 1 m height on the *Hymenaea* trunk; note abundant specimens of four ant morphotypes (four black arrows) of very diverse sizes which became trapped before skeletonization of the gecko carcass. (**C**) Gecko carcass on sticky trap (down right) placed on the tree NCR 25 at 0 m height on the *Agathis* trunk; note abundant specimens of at least three fly morphotypes of Muscidae, Dolichopodidae and Phoridae (three white arrows) of very diverse sizes which became trapped when attracted by the gecko carcass and three ants (black arrow) visiting the carcass but not trapped; however, the smaller morphotype (Phoridae) is also very abundant in the main part of the other sticky traps in this station with or without trapped geckos. (A and B) from campaign 2013 in Ambahy community (Nosy Varika, Mananjary region) (20° 46′ S, 48° 28′ E) and from campaign 2017 in Andranotsara forest, 40 km south of Sambava city (14° 38′ S, 50° 12′ E), respectively (both areas at the east coast of Madagascar; more information in Delclòs et al., 2020). (C) from campaign 2016 in Faux Bon Secours forest, South of New Caledonia (22° 10′ S, 166° 41′ E). Trap width 7.35 cm in (**A**), 7.50 cm in (**B**), 10.00 cm in (**C**).

Lizards were also accidentally trapped in abundance (due to the strong adhesive used in the trap) in actualistic experiments. Plant remains and arthropods were collected using yellow sticky traps in Madagascar on the trunks of *Hymenaea verrucosa* Gaertner, 1791 and New Caledonia on the trunks of *Agathis lanceolata* Warburg, 1900. We observed that abundant arthropod specimens were attracted to the trapped lizards, including several ant morphotypes of different sizes and three different flies of Muscidae, Dolichopodidae and Phoridae (Fig. 7). In these actualistic observations, ants were noticeably more abundant than flies in both resin-producing forests.

## Discussion

The bioinclusions oryctocenosis or taphocoenosis of a single amber piece can be heterogeneous (containing a mixture of specimens from two or more different kinds of biocoenosis) and/or homogenous, whereby most amber pieces contain syninclusions in heterogeneous taphocoenosis^25^. Because most resin pieces grew from the accumulation of distinct resin flows, the bioinclusions could be from asynchronous events^11,37^. This important taphonomic aspect has been little discussed. For that reason, a potential biological relationship or a behaviour has to be treated with caution because the recorded organisms did not necessarily interact in life^43^. In this regard, the presence together in an amber piece of sets of syninclusions (eusyninclusions), that are parasyninclusions in respect each to other, implies much more relevant and varied palaeoecological data available. The sequence of sets of eusyninclusions could provide exceptional information, for example of periodic ecologic phenomena^38^. For these reasons, some amber pieces must be studied describing their “stratigraphy” or sequence of layers determining the oldest and younger layers and the content of eusyninclusions of each, as well as other interesting taphonomic evidence, if present. This distinction of both types of syninclusions allows more accurate inference of the spatial and temporal relationships between different bioinclusions, following the aim of the term syninclusion defined by Koteja^29^ and enhancing it. The presence of bioinclusions in different layers (consecutives, close, distanced) provide indirect, but relevant evidence of temporal distance between parasyninclusions, but not relevant distance in terms of space, because they could be considered the same in a centimetric scale in respect to the resiniferous tree. However, the presence of bioinclusions in different layers introduces a key circumstance in the study of close organismal relationships, for example between a pollinator insect and pollen grains in the same amber piece, because both types of syninclusions present in consecutive layers could be very close spatially to each together, but not temporally.

Cretaceous amber pieces containing rich sets of eusyninclusions are not rare, however those evidencing key palaeoecological data are uncommon. Vertebrate remains and/or vertebrate bodies included in amber related to eusyninclusions are likewise uncommon^44^, and can lead to important information about the process of decomposition in the past. We conducted herein the study of three amber pieces from Myanmar of the latter type. This small amount is exceptional, because lizards are extremely rare in amber and only few are available to the science^45,46^. This rareness is not expected considering that modern lizards climb trees to look for food and to sun themselves, and potentially could be trapped in resin emissions. Some reasons for their rareness are probably their larger body sizes that generally allow them to escape from sticky resin emissions and that not all lizards look for their food sources on the trees^12^.

In the studied amber piece GRS-Ref-28627, except for the rare spiders, all other bioinclusions are "aerial" inhabitants and not typical part of the forest litter, that would be with the presence of certain debris and arthropod groups such as Isopoda, Myriapoda, Acari, Araneae, Pseudoscorpionida or Collembola^47^. Thus, we can consider this as an "aerial amber piece". With "aerial amber", we mean an amber piece, generally translucent and "clean" of detritus, containing flying insects, other arthropods, or other animals living on the tree trunk, or organismal elements reaching the resin through the help of the wind^11^ (small leaves, plan trichomes, etc.) Typically, this kind of amber contains evidence of gravity generally in the form of inner concentric banding patterns corresponding to desiccation surfaces in trunk and branches of successive resin flows that came in a relatively short time and gave the possibility to trap organisms and dry up before the subsequent resin flow^11^. The resin that caught the lizard and the other organisms in amber piece GRS-Ref-28627 was produced on the tree trunk high on the resiniferous tree. Most probably, species of *Oculudentavis* inhabited and fed on small arthropods on the tree trunk and branches. It is unlikely that *Oculudentavis naga* got caught in the resin wanting to eat the big beetle on layer 10 (Figs. 2, 4), because it got stuck two resin layers before the *O. naga* did. Thus, these are not eusyninclusions with respect to each other.

Layer 12 was formed with an “open window” (showing a small area not well covered by the resin flow) above the neck of the *Oculudentavis* carcass, from which the smell of decomposition could spread, and that could probably have attracted a heterogeneous swarm of flies. The assemblage strongly suggests that it was composed by necrophagous and predaceous flies (see below a detailed argumentation). We here propose the term "necrophagous trap" for this taphonomic phenomenon, which falls into what is defined as a conservation trap, a concept recently reviewed^48^. The term and concept "necrophagous trap" could also be used for other kinds of palaeontological outcrops such as those in tar pits and pseudokarstic pits, involving insects and/or vertebrate scavengers and carnivores^7,49^. These traps can be considered as ancient natural bait traps.

Experiments with modern lizards indicate that the muscles are liquefied and degrade rapidly by the action of microorganisms and fly larvae, and, in two days, the smell of ammonia is noticeable, attracting decomposer organisms, including further flies^50^. These decompose the soft parts of the lizards in about 6 days^16^. In our case, the flies visiting the exposed portion of the carcass easily became trapped when they contacted the sticky resin surface with any body part, facilitated by the abundance of individuals and their competition for food. A subsequent resin flow covered and preserved the "necrophagous trap" with abundant phorids and empidoids as a frozen record of this specific moment of the carrion association. Exceptional preservation is frequent in amber but is not the rule, since preservation can be quite variable depending e.g. on chemical components and gut microbiota^51,52^. The flies around the neck, trapped as eusyninclusions of the layer 12, are poorly preserved compared to the parasyninclusions in the piece. The bodies appear well articulated, but with some degree of deformation, with the cuticle somewhat degraded looking transparent, and most of them are collapsed, especially the specimens closest to the neck. Most likely, the poor preservation is due to the action of ammonia that, in contact with water molecules, can affect arthropods and other kind of animals as for example squids and cuttlefish at a cellular and subcellular level limiting its preservation potential^53,54^. Another explanation, not exclusive, is the long time that the trapped flies were exposed to dehydration in virtually aerial conditions before the subsequent resin flow that covered them. In general, three-dimensionally complete insects preserved in amber correspond to individuals completely embedded in resin in a short time and thus deceased within the resin^52^.

The study of necrophagous flies focuses more on species of forensic importance and less on their role as decomposers in the forests. Thus, most of the information is provided from such kind of studies. The diversity of necrophagous insects varies according to geographical areas, climates, number of individuals, hosts, size of the hosts, and also through time^2^, and depending on their preference for a given stage of decomposition, colonization occurs in a chronological sequence^3^. Today, the most common families reported as necrophagous are dipterans of Callophoridae, Sarcophagidae, and Muscidae, which first colonize dead animals if their large size allows it^2^. None of these three families had yet evolved in the Cretaceous^55^. However, many other families are also well known as carcasses visitors such as Phoridae, Chloropidae, Drosophilidae, Micropezidae, Otitidae, Sepsidae, and Sphaeroceridae, which are specifically associated with carrion of lizards^16^. The small necrophagous dipterans commonly appear when the viscera decompose and the fat of the corpse turns rancid^2^. This is not surprising for Phoridae, since the small scuttle flies can dominate the carcass fauna appearing at the early stages of decay, especially when larger flies cannot gain access^56^. Among them, in modern fauna, is the megadiverse genus *Megaselia*, which contains, among others, facultative scavenger species^57^ breeding in a large diversity of animals, including lizards and arthropods in wet tropical forest as well as in temperate forest^16,18^. This genus does not appear in the fossil record until the Cenozoic, with the oldest *Megaselia* specimen from the Fushun amber, *M. multopalputa* Hong, 1981, however^58–60^.

Tomberlin et al.^61^ mentioned two intervals in the colonization of dead animals by necrophagous arthropods. In the pre-colonization interval, they recognized the exposure time, the detection time, and the acceptance. The post-colonization interval comprises consumption and dispersal time^61^. The time intervals are different depending on the species. Neither eggs nor complete larvae were present in our studied samples, and only adults were observed. The resin in layer 13 filled out parts on the lizard neck where the soft parts were already consumed, as is well observed in the synchrotron images (Fig. 1A). Based on this information, the most possible scenario to explain the presence of these two kinds of flies is that the Phoridae swarm was attracted by the dead lizard, which was only partly embedded in resin and the exposed part was already in a rancid phase. Phorids were trapped, in the exposed surface of the layer 12, during the consumption phase of their post-colonization interval. This explanation better clarifies the presence of the empidids since most dance flies are general predators, most of them on living insects, but others also feeding on dead insects^62^. Empidids are known to form mating and non-mating swarms^63^. Most of the Empidoidea in the amber piece are females, however, and it is not possible to establish if the swarm was a mating or a non-mating event. Conversely, "phorids amber swarms" were argued to be related to the massive attraction to dead and decaying organic material^23^.

There are more specimens of lizards preserved in Burmese amber that were entirely covered by the resin (total encapsulation), and they are not part of an association like this (Figs. 5, 6). With certainty, the phorids that had this trophic strategy could find many dead lizards exposed to the air, for example in the litter far from resin emissions. However, this studied case is very peculiar because it originated by a resin flow that left a large part of the lizard body inaccessible to the phorids or other kind of flies, but at the same time constituting a necrophagous trap around the temporarily exposed portion.

In actualistic experiments with sticky traps, a non-natural entomological trap that correlates with resin^12,22^, we observed dipterans and ants trapped around the trapped lizards (see figuration in Solórzano Kraemer et al.^12^ and in the present work Fig. 7). Ants walk on the sticky traps and on resin to reach the carcasses having more probability than the flies of getting trapped in the sticky traps. However, the phenomenon of lizard and ants together in a single amber piece is not described from Cretaceous amber, and was not seen in the 21 Cenomanian pieces revised in the Peretti Museum Foundation. Conversely, the lizard genus *Anolis*, well known in Miocene Dominican and Mexican ambers^64^, has been reported to appear with flies and ants in single pieces^65,66^ and there is no reason to assume ants would have less capacity for preservation than the flies. Unfortunately, most of the works on lizards in Miocene and Eocene ambers do not report syninclusions, so the number of cases could be higher.

In a study by Cornaby^16^ in a tropical dry and wet forest in Costa Rica, larvae and adults of 172 arthropod species were identified as scavengers on dead lizards and toads. Dipterans and ants feeding on lizards were represented by hundreds and thousands in the dry and wet forests, respectively; however, ants were more important in the wet forest^16^.

Necrophagy is often seen in social ants and is thought to be beneficial to the colony as a whole, as it helps to reduce the risk of disease and infection, and can also provide a source of nutrition for the colony. Additionally, necrophagy can help to strengthen the social bonds between ants, as it is often seen as a form of altruistic behaviour^67,68^.

Ants are predatory insects that succeeded from the Cretaceous until today to become one of the most abundant insects in tropical forests thanks to their ability to develop social links and to live in communities^69^. Today, the effects of ants on nutrient decomposition, soil turnover, and perturbation is immense due to the extremely high abundance at a global level, being larger than the biomass of bids or mammals^70^. However, they are rather rare in Cretaceous deposits, increasing continually in abundance in Eocene and Miocene amber deposits^69,71,72^.

In conclusion, the present work describes the presence as eusyninclusions of flies in close relation to lizard bodies in Cenomanian amber; however, Cretaceous ants have never been recorded in similar circumstances. This could be due to (1) a limited amount of revised material (even if we here revised probably one of the largest lizard amber collections) or (2) circumstances suggesting that the ants would not yet have a trophic or foraging strategy to search for vertebrate corpses and to eat carrion, unlike in the current myrmecofauna. The second hypothesis is congruent with the relatively rare evidence of ants that preserved instances of social behaviour during the Cretaceous^69^.

## Material and methods

### Amber pieces

The piece with the number GRS-Ref-28627 containing the *Oculudentavis naga* holotype^39^ was recovered in the Aung bar mine (26° 09′ N, 96° 34′ E), Tanai Township (Myitkyina District, Hukawng Valley, Kachin Province), northern Myanmar. The piece GRS-Ref-28631 includes an incomplete lizard and some phorids and other Brachycera flies, and was recovered from the Zee Phyu Kone mine, just next to Aung bar mine. The piece GRS-Ref-060905 contains a part of a lizard and a lot of phorid wings, and it was recovered in the Hkamti mine. According to Shi et al.^73^, the age of the amber in the Hukawng Valley is 98.8 ± 0.6 Ma and thus of the Cenomanian Period. The amber from the Hkamti mine has been dated as 101.5–94 Ma based on foraminifera species *Douvilleiceras mammillatum* (Schlotheim, 1813), *Mortoniceras* (*Mortoniceras*) *fallax* (Breistroffer, 1940), and *Hoplites* (*Hoplites*) *mirabiliformis* Spath, 1925, obtained from the amber-bearing layer (unpublished data from A. Peretti). The same amber deposit has been dated as 110 Ma according to zircon analyses^74^. Authenticity and origin of Burmese amber samples follow Muza et al.^75^ and was applied to the samples studied here along with additional analysis of the amber matrix^76^.

The criteria we used to define resin layers is quite simple, namely the natural limits that were created by the drying of the resin before the next resin flow came. The layer limits in the external surface of the amber pieces were well distinguished with the help of UV fluorescence.

The analysis of the amber samples was made during the symposium organized by the Peretti Musem Foundation in December 2019 in Bangkok, Thailand (https://www.youtube.com/watch?v=JZK0sYlyckc), and in a second workshop in Meggen, Switzerland (2022).

### Sticky trap material

Sticky traps were collocated on the trunks of *Hymenaea verrucosa*, a resin-producing angiosperm tree growing in Madagascar. In a second experiment, sticky traps were placed on the trunks of *Agathis lanceolata*, a resiniferous gymnosperm tree growing in New Caledonia. The goal of these experiments was to collect arthropods; however, also lizards were accidentally trapped.

Sampling material were done with permits from the Government of Madagascar and New Caledonia under the respective numbers: Sampling Permit N. 160/13 and N. 192/17; exportation of samples N. 186 N-EA10/MG13 and N. 252N-EA10/MG17 in Madagascar; and sampling and exportation N. 3021-2016/ARR/DENV in New Caledonia.

The sticky traps were active, collecting organisms for eight days. After that, the glue of the sticky sheets was dissolved with gasoline to get their contents and be transferred to alcohol immediately (see^12^ for more details).

### Imaging

The photographs and Z-stacks images were performed under a Keyence VHX-7100 microscope, using Keyence VHX-E objectives. The piece number GRS-Ref-28627 is documented here as having two surfaces, sides A and B. These sides are illustrated in Fig. 4A (side A) and Fig. 4C (side B). The UV-Fluorescence images were acquired using a standard Wood Lamp, UVGL 58 model–UVP Analytik Jena, equipped with long-wave (365 nm) and short-wave (254 nm), 8-Watt lamps. Images were acquired with a Canon EOS R camera using a 24–105 mm lens, placing the amber samples in a dark box. Figures were performed using Adobe Photoshop software (CS6, version; 13.0 www.adobe.com).

### Ethics statement

Export permit for all pieces is 124,627. The paper trail of GRS-Ref-28627^39^ is valid for GRS-Ref-28631. Piece GRS-Ref-060905 was found in 2014 in Hkamti, and was bought with certification and origin declaration from China imported into Hong Kong in 2021 (a declaration of the supplier is available). All three pieces are deposited in the Peretti Museum Foundation and were acquired by A. Peretti. None of the pieces have been directed to support conflict in Kachin. Acquisition and scientific work were done following the Myanmar laws. Peretti Museum Foundation law mandatorily guarantees under Swiss law that the foundation's inventory with GRS Reference numbers can never be lost to science. For a complete report of the ethic statements, see^39,77^. Detailed information of the ethical acquisition of amber pieces can be found in Bolet et al.^39^.

The Malagasy Institute for the Conservation of Tropical Environments (ICTE/MICET) and the Direction de l’Environnement (DENV) Province Sud from Nouvelle Calédonie approved the sticky traps experimental protocols. All methods were carried out in accordance with relevant guidelines and regulations and study was reported in accordance with the arrive guideline.

## Data Availability

All data needed to evaluate the conclusions in the paper are present in the paper. Correspondence and material related to this paper may be requested from Mónica M. Solórzano Kraemer (monica.solorzano-kraemer@senckenberg.de), and Enrique Peñalver (e.penalver@igme.es). All amber samples studied are deposited in the collection of the Peretti Museum Foundation in Meggen (Switzerland) (https://www.pmf.org/).
